# Postnatal Hyperoxia Exposure Differentially Affects Hepatocytes and Liver Haemopoietic Cells in Newborn Rats

**DOI:** 10.1371/journal.pone.0105005

**Published:** 2014-08-12

**Authors:** Guya Diletta Marconi, Susi Zara, Marianna De Colli, Valentina Di Valerio, Monica Rapino, Patrizia Zaramella, Arben Dedja, Veronica Macchi, Raffaele De Caro, Andrea Porzionato

**Affiliations:** 1 Department of Pharmacy, University “G. d’Annunzio” Chieti-Pescara, Chieti, Italy; 2 Department of Medicine and Ageing Sciences, University “G. d’Annunzio” Chieti-Pescara, Chieti, Italy; 3 Institute of Molecular Genetics CNR, Unit of Chieti, Chieti, Italy; 4 Neonatal Intensive Care Unit, Women’s and Children’s Health Department, University of Padova, Padova, Italy; 5 Department of Molecular Medicine, University of Padova, Padova, Italy; University College London, United Kingdom

## Abstract

Premature newborns are frequently exposed to hyperoxic conditions and experimental data indicate modulation of liver metabolism by hyperoxia in the first postnatal period. Conversely, nothing is known about possible modulation of growth factors and signaling molecules involved in other hyperoxic responses and no data are available about the effects of hyperoxia in postnatal liver haematopoiesis. The aim of the study was to analyse the effects of hyperoxia in the liver tissue (hepatocytes and haemopoietic cells) and to investigate possible changes in the expression of Vascular Endothelial Growth Factor (VEGF), Matrix Metalloproteinase 9 (MMP-9), Hypoxia-Inducible Factor-1α (HIF-1α), endothelial Nitric Oxide Synthase (eNOS), and Nuclear Factor-kB (NF-kB). Experimental design of the study involved exposure of newborn rats to room air (controls), 60% O_2_ (moderate hyperoxia), or 95% O_2_ (severe hyperoxia) for the first two postnatal weeks. Immunohistochemical and Western blot analyses were performed. Severe hyperoxia increased hepatocyte apoptosis and MMP-9 expression and decreased VEGF expression. Reduced content in reticular fibers was found in moderate and severe hyperoxia. Some other changes were specifically produced in hepatocytes by moderate hyperoxia, i.e., upregulation of HIF-1α and downregulation of eNOS and NF-kB. Postnatal severe hyperoxia exposure increased liver haemopoiesis and upregulated the expression of VEGF (both moderate and severe hyperoxia) and eNOS (severe hyperoxia) in haemopoietic cells. In conclusion, our study showed different effects of hyperoxia on hepatocytes and haemopoietic cells and differential involvement of the above factors. The involvement of VEGF and eNOS in the liver haemopoietic response to hyperoxia may be hypothesized.

## Introduction

Hyperoxic ventilation is frequently involved in neonatal intensive care units for treatment of respiratory distress syndrome and pulmonary hypertension. However, many authors recommend limitation of hyperoxia exposure in the newborn period due to increased awareness about its noxious effects [1]–[3]. Premature newborns, in particular, are known to be more susceptible to oxidative stress due to immaturity of the antioxidant system [4]–[5] and due to deficiency of antioxidant precursors in parenteral nutrition [6]. The role of hyperoxia in the pathogenesis of bronchopulmonary dysplasia and retinopathy of prematurity has been widely studied but the effects of neonatal hyperoxia on the other organs have not yet been fully considered.

Some authors suggested that hyperoxic effects on liver may contribute to lung injury due to hyperoxia exposure [7]–[10] and that the liver may possibly represent a therapeutic target for patients with hyperoxia-induced lung injury [11]. In particular, it has been suggested that molecules released by the liver in response to hyperoxia may enter the bloodstream and produce responses in the lung [10]. In the literature, some reports are present about the effects of hyperoxia on the expression and activities of hepatic proteins in rats and mice, with particular reference to metabolic activities. Increased expressions of Cyp1a1/Cyp1a2, arginase I, 5-lipoxygenase, cyclooxygenases-2, iNOS, mitochondrial enoyl-CoA hydratase I and heme-oxygenase-1 have been reported in rodent liver in different types of hyperoxia exposure [7], [9]–[15]. Conversely, hyperoxia exposure has been reported to reduce the expression of 10-formyltetrahydrafolate dehydrogenase, alpha-tochopherol transfer protein and other cytochrome isoforms [15]–[17]. There are no data, instead, about hyperoxia-induced modulation in liver of growth factors and signaling molecules which are known to be involved in other tissues, such as Vascular Endothelial Growth Factor (VEGF), Matrix Metalloproteinases (MMPs), Hypoxia-Inducible Factor-1α (HIF-1α), endothelial Nitric Oxide Synthase (eNOS), and Nuclear Factor-kB (NF-kB).

In rodents, the bone marrow becomes active in haemopoiesis after birth but liver and spleen maintain this function at least in the first postnatal period. It is known that in early embryo the yolk sac represents the primary site of haemopoiesis. Haematopoietic stem cells then colonize the umbilical cord, the aorta-gonad-mesonephros region and later the embryonic liver. The liver is usually considered to cease its action in haematopoiesis soon after birth but it maintains haematopoiesis at low levels also during adulthood [18]. Hypoxia is a known inducer of medullary and extramedullary erythropoiesis but no data are available about the effects of hyperoxia in liver haematopoiesis.

The aim of the present study was to evaluate the hepatic effects of neonatal exposure to chronic moderate (60% O_2_) and severe (95% O_2_) hyperoxia, with particular reference to the main factors involved in response to hyperoxia. Hyperoxia exposure has been reported to induce proliferative responses in foci containing stem cells, such as neurogenic sites [19], so that particular attention was also put on liver haemopoiesis and in possible mechanisms involved.

## Materials and Methods

### Animals and experimental procedure of hyperoxia exposure

Female wild-type Sprague-Dawley rats (Harlan, Udine, Italy) and their offspring were housed and handled in strict accordance with the recommendations in the Guide for the Care and Use of Laboratory Animals of the National Institutes of Health. The study was approved by the Italian Ministry of Health (Permit Number: 220/2009-B). Experimental procedures of hyperoxia exposure have been detailed in previous works [19]–[25]. Briefly, the study was conducted on male or female rat pups kept together with their nursing mother in conventional facilities. Mothers and litters were placed in clear polished acrylic chambers provided with software enabling a continuous monitoring of O_2_ and CO_2_ (BioSpherix, OxyCycler model A84XOV, Redfield, NY). Animals were maintained under standardized conditions of light (12∶12 h light-dark cycle, illumination onset 07.00 a.m.) at 24°C. After term gestation, the newborn rats were randomly distributed between the following experimental groups: a) newborn rats (n = 6; 3 male, 3 female) raised in ambient air for 2 weeks; b) newborn rats (n = 6; 3 male, 3 female) continuously exposed to 60% oxygen for 2 weeks; c) newborn rats (n = 6; 3 male, 3 female) continuously exposed to 95% oxygen for 2 weeks. Each experimental group comprised a balanced sample of rats across litters, i.e., two rats (one male and one female) from each of three litters, in order to limit possible interfering effects due to litter differences. According to the literature on matter [26]–[27], the nursing dams of each group were rotated every one day to prevent any negative effect of hyperoxia on nursing. The offspring were well nursed and the dam rotation did not exert any negative influence on the pups. The above O_2_ percentages were considered as they are the most frequently used models of hyperoxia exposure in the literature. In particular, 60–65% and 95% O_2_ at sea level are usually defined as moderate and severe hyperoxia, respectively [26]–[28]. At the experimental endpoints animals were sacrificed with Nembutal (40 mg/kg). Direct analyses of tissue oxygen partial pressure were not performed in the present study but increased levels of oxygen and hemoglobin oxygen saturation have already been demonstrated in previous analogous experimental protocols [29]–[31]. Moreover, hyperoxic breathing has been reported to increase portal pO_2_ and portal venous oxygen saturation [32]. Liver was excised from each rat, washed in phosphate-buffered saline (PBS) and cut into two blocks which have been fixed in 10% phosphate-buffered formalin or frozen at −80°C.

### Light microscopy analysis and immunohistochemistry

Tissues were fixed in 10% phosphate-buffered formalin for 72 h. Each tissue block was dehydrated in a series of alcohol solutions of 50%, 70%, 80%, 95%, and 99% and then in xylene. Samples were then paraffin-embedded and cut into 5 µm-thick sections. Sections were de-waxed (xylene and alcohol in progressively lower concentrations), rehydrated and processed for haematoxylin-eosin and Silver staining (Bio Optica, Milano, Italy), according to manufacturer protocols, and for anti- Ki-67, CD34, CD45, VEGF, eNOS, NF-kB and HIF-1α immunohistochemical analyses.

As it regards immunohistochemical protocols, sections were incubated in 0.3% hydrogen peroxide for 10 min at room temperature, to remove endogenous peroxidase activity. To block non-specific background staining slides were incubated with UltraVision Protein Block (Lab Vision Thermo, CA, USA) for 10 minutes at room temperature.

Anti-CD34 immunohistochemical staining was preceded by permeabilization with proteinase K (DakoCytomation, S3020) for 5 min at room temperature. Anti-CD45 immunohistochemical reaction was preceded by antigen unmasking by heating with 10 mM sodium citrate buffer, pH 6.0, at 90°C for 10 min. For the other immunohistochemical stainings antigen unmasking was not necessary.

The primary antibodies used were the following: rabbit monoclonal anti-ki-67 (M3062, Spring Bioscience, Pleasanton, CA, USA); rabbit polyclonal anti-VEGF (sc-152, Santa Cruz Biotechnology, Santa Cruz, CA, USA), anti-NF-kB (sc-109, Santa Cruz Biotechnology), and anti-eNOS (sc-654, Santa Cruz Biotechnology); mouse monoclonal anti-CD34 (sc-7324, Santa Cruz Biotechnology), anti-CD45 (Biocare Medical, clone FL3A11, purified 5450-P) and anti-HIF-1α (sc-53546, Santa Cruz Biotechnology). Primary antibodies were applied for 90 minutes at room temperature. Anti-Ki-67 antibody was diluted 1∶200 in PBS; all the other antibodies were diluted 1∶100 in PBS. Slides were incubated with Primary Antibody Enhancer (Lab Vision Thermo, CA, USA) for 10 minutes at room temperature, then with HRP Polymer (Lab Vision Thermo, CA, USA) for 30 minutes at room temperature. The immunohistochemical reactions were revealed with DAB Plus Chromogen (Lab Vision Thermo, CA, USA). Peroxidase reaction was developed using diaminobenzidine (DAB) chromogen and nuclei were counterstained with haematoxylin. Lastly, sections were dehydrated, cleared with xylene (J. T. Baker, Deventer, Holland), and mounted in Eukitt (O. Kindler GmBH, Freiburg, Germany). Negative controls were performed by omitting the primary antibody. Samples were then observed by means of Leica DM 4000 light microscopy (Leica Cambridge Ltd., Cambridge, UK) equipped with a Leica DFC 320 camera (Leica Cambridge Ltd.) for computerized images.

### Terminal-deoxinucleotidyl-transferase-mediated dUTP nick end-labeling analysis

Terminal-deoxinucleotidyl-transferase-mediated dUTP nick end-labeling (TUNEL) is a method of choice for a rapid identification and quantification of apoptotic cells. DNA strand breaks, yielded during apoptosis, can be identified by labeling free 3′-OH termini with modified nucleotides in an enzymatic reaction. Paraffin embedded tissue sections were de-waxed and rehydrated. All steps were realized with FragEL DNA fragmentation Detection Kit according to the manufacturer’s instructions (Calbiochem Merck, Cambridge, MA, USA). After two rinses in PBS, tissue slides were dehydrated, mounted by using a permanent media and analyzed by light microscope. Three slides from each animal were assessed, apoptotic cells count was performed on ten fields per slide. Negative controls were performed by omitting the incubation in the presence of the enzymatic mixture, positive control was performed by treating one slide with DNAse I.

### Computerized morphometry measurements and image analysis

After digitizing the images deriving from immunohistochemically and Silver stained sections, morphometric softwares were used for image analysis as follows. The percentage area of haemopoiesis was calculated with ImageJ software, a public domain Java image processing program created at the Research Services Branch, National Institute of Health, Bethesda, MD, USA (http://rsb.info.nih.gov/ij). Analyses were performed on 1132 µm×841 µm fields. The haemopoietic foci were manually delineated and the ratio between their areas and the area exhibited by the whole section was calculated and expressed in form of percentage.

The amount of reticular fibers (Silver staining) and eNOS expression were evaluated in terms of percentage area on 271 µm×185 µm fields with QWin Plus 3.5 software. In each field, the areas stained in brown, with immunohistochemistry, and black, with Silver staining, were automatically measured after definition of the color interval at the histograms of distribution of hue, saturation and brightness. The ratio between the positive area and the area exhibited by the whole section was calculated and expressed in form of percentage.

Anti-VEGF, NF-kB, and HIF-1α immunostainings, together with TUNEL reaction, were morphometrically evaluated in terms of percentages of positive nuclei on 271 µm×185 µm fields. In each field, the total number of hepatocyte nuclei and the number of positive nuclei were directly counted. The percentages of positive nuclei were derived by dividing the number of positive nuclei by the total number of nuclei and multiplying by 100.

Mean morphometric values for each parameter and each case were derived from ten fields taken from each of three slides (total number of fields examined = thirty). Fields were chosen in each section through systematic and uniformly random sampling of the fields of vision through a pre-defined XY step, i.e., randomly choosing the first field in a section and then identifying the other fields by constant predefined movements along the X and Y axis. This procedure is usually followed in stereology to allows unbiased, efficient and precise estimates [19].

### Western blotting analysis

Liver lysates (20 µg) were electrophoresed and transferred to nitrocellulose membrane. Nitrocellulose membranes, blocked in 5% non-fat milk, 10 mmol/L Tris pH 7.5, 100 mmol/L NaCl, 0.1% Tween-20, were probed with mouse monoclonal anti-β-tubulin antibody (T4026, Sigma-Aldrich, St Louis, MO, USA) (primary antibody dilution 1∶1000), mouse monoclonal anti-MMP-9 (catalog number: IM37, Calbiochem Merck, Cambridge, MA, USA) (primary antibody dilution 1 mg/ml) and rabbit polyclonal anti-e-NOS antibodies (Santa Cruz Biotechnology, Santa Cruz, CA, USA) (primary antibody dilution 1∶200) and then incubated in the presence of specific enzyme conjugated IgG horseradish peroxidase. Immunoreactive bands were detected by ECL detection system (Amersham Int, Buckinghamshire, UK) and analysed by densitometry. Densitometric values, expressed as Integrated Optical Intensity (IOI), were estimated in a CHEMIDOC XRS system by the QuantiOne 1-D analysis software (BIORAD, Richmond, CA, USA). Values obtained were normalized basing on densitometric values of internal β-tubulin.

### Statistics

Statistical analysis was performed with GraphPad Prism 5 software applying the analysis of variance (one-way ANOVA and Bonferroni test). Results are expressed as mean ± SD. Values of p<0.05 were considered statistically significant.

## Results

**Figure 1:**
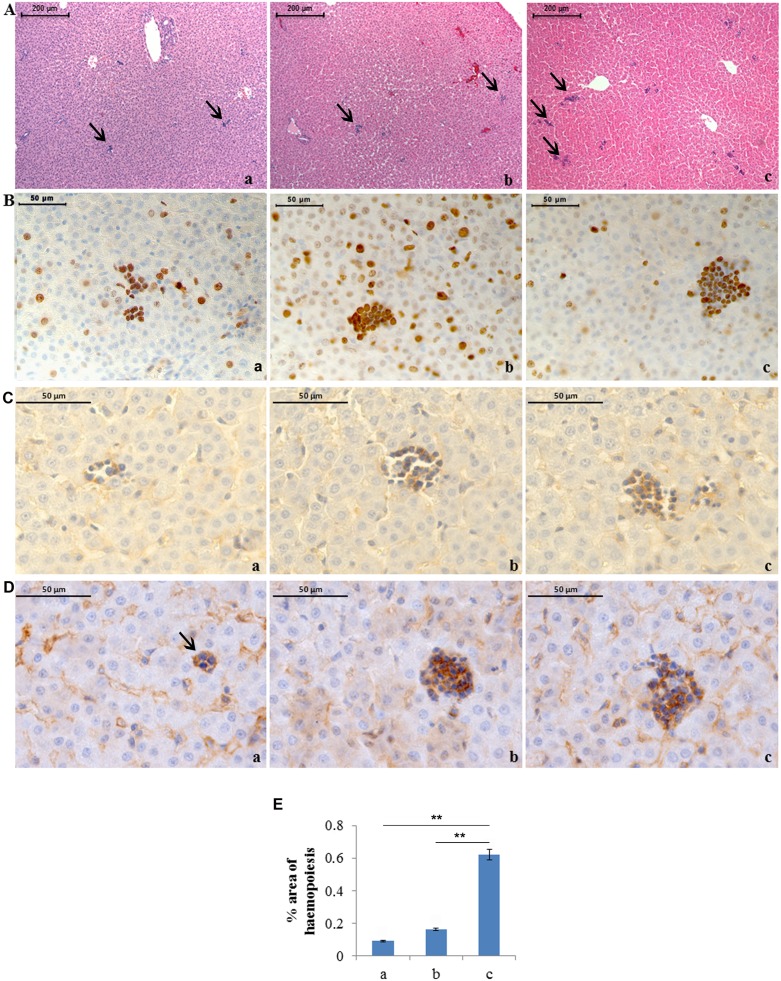
Severe hyperoxia increases liver haemopoiesis. A) Haematoxylin-eosin staining of neonatal rat livers exposed to moderate and severe hyperoxia. a) ambient air; b) 60% hyperoxia; c) 95% hyperoxia. Arrows indicate haemopoietic foci. Scale bar: 200 µm, magnification 10x. B) Anti-ki67 immunohistochemistry showing diffuse positivity of haemopoietic cells. Scale bar: 50 µm, magnification 40x. C) Anti-CD34 immunohistochemistry showing diffuse positivity of haemopoietic cells. Scale bar: 50 µm, magnification 63x. D) Anti-CD45 immunohistochemistry showing diffuse positivity of haemopoietic cells. Scale bar: 50 µm, magnification 63x. E) Graphic representation of the mean values of the percentage area of liver haemopoietic foci (±SD) determined in ten fields for each of three slides per sample. ** = p<0.01.

Sex differences were not found in the analysis of the various parameters. Significantly increased areas of haemopoiesis were found through morphometry in livers of rats exposed to severe hyperoxia with respect to normoxia and moderate hyperoxia (Fig. 1). Anti-Ki67 immunohistochemistry showed diffuse positivity of haemopoietic cells in all groups; haemopoietic cells in samples exposed to hyperoxia appeared larger. Positivity of the above foci for anti-CD34 and -CD45 immunohistochemistries confirmed the haemopoietic nature of the cells (Fig. 1).

**Figure 2:**
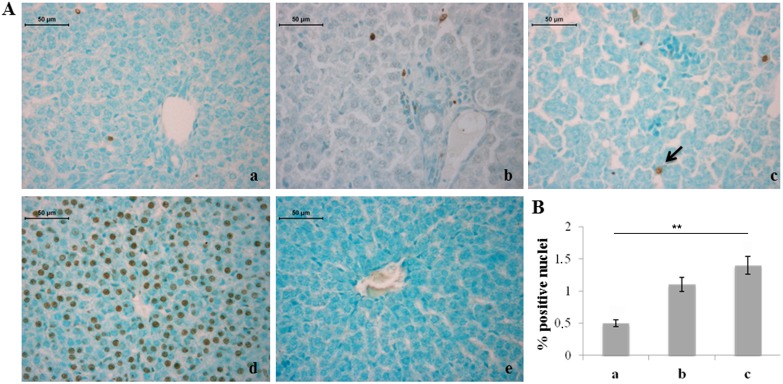
Severe hyperoxia increases hepatocyte apoptosis. A) TUNEL detection of apoptotic nuclei (arrow) in neonatal rat livers exposed to moderate and severe hyperoxia. a) ambient air; b) 60% hyperoxia; c) 95% hyperoxia; d) positive control; e) negative control. Scale bar: 50 µm, magnification 40x. B) Graphic representation of the percentage of TUNEL positive nuclei (±SD) determined by direct visual counting of ten fields for each of three slides per sample. ** = p<0.01.

As it regards possible noxious effects of hyperoxia on the liver tissue, TUNEL analysis showed statistically significant increase in the percentage of apoptotic hepatocytes in rats exposed to 95% oxygen compared to rats exposed to normoxia (Fig. 2). Different percentages of TUNEL positivity were not found in haemopoietic foci of the three experimental groups (data not shown).

**Figure 3:**
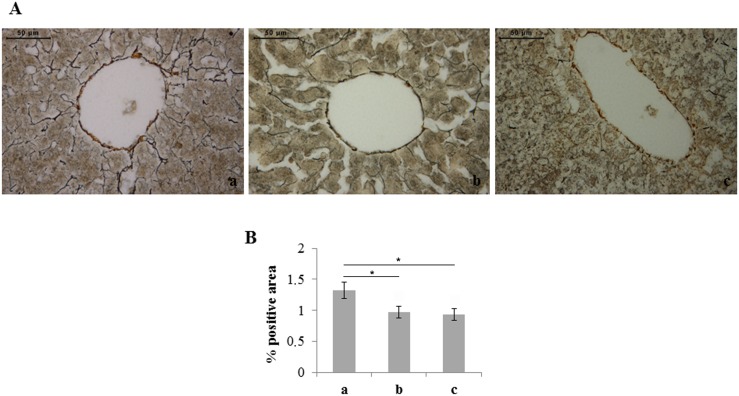
Moderate and severe hyperoxia reduces the amount of reticular fibers in rat liver. A) Silver staining of neonatal rat livers exposed to moderate and severe hyperoxia. Reticular fibers are labeled by black stain. In livers of rats exposed to moderate and severe hyperoxia a lower amount of reticular fibers is visible. a) ambient air; b) 60% hyperoxia; c) 95% hyperoxia. Scale bar: 50 µm, magnification 40x. B) Graphic representation of the percentage area of reticular fibers (±SD); densitometric analysis determined by quantifying thresholded area for black color in ten fields for each of three slides per sample. * = p<0.05.

Silver staining, which selectively stains reticular fibers in black, showed a reduced amount of reticular fibers in rats exposed to both moderate and severe hyperoxia with respect to unexposed ones (Fig. 3).

**Figure 4:**
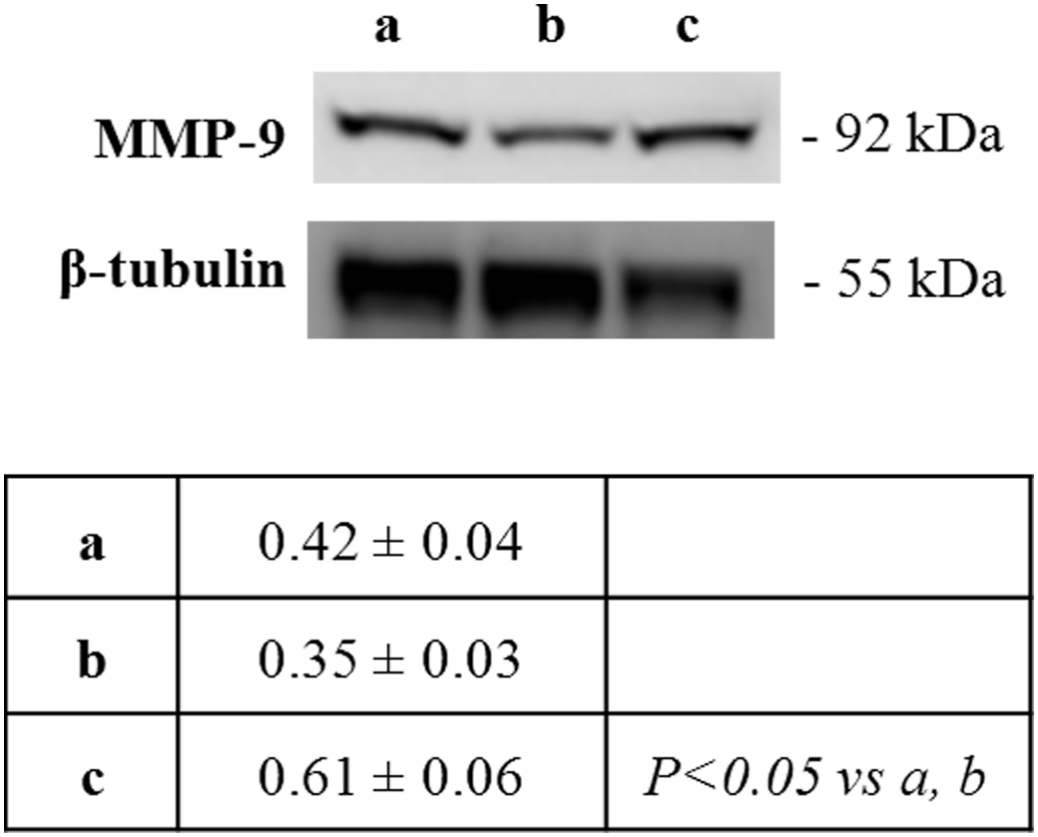
Severe hyperoxia increases liver expression of MMP-9. Western blotting analysis of MMP-9 expression in neonatal rat livers exposed to moderate and severe hyperoxia. a) ambient air; b) 60% hyperoxia; c) 95% hyperoxia. Each membrane has been probed with anti-tubulin antibody to verify loading evenness. Data are the densitometric measurements of protein bands expressed as mean values of Integrated Optical Intensity (IOI) (±SD).

Due to the above changes in components of the extracellular matrix, we then focused our attention on MMP-9, analysed through Western blot. Increased liver expression of this molecule was observed in rats exposed to severe hyperoxia compared to moderate hyperoxia-exposed and unexposed ones (Fig. 4).

**Figure 5:**
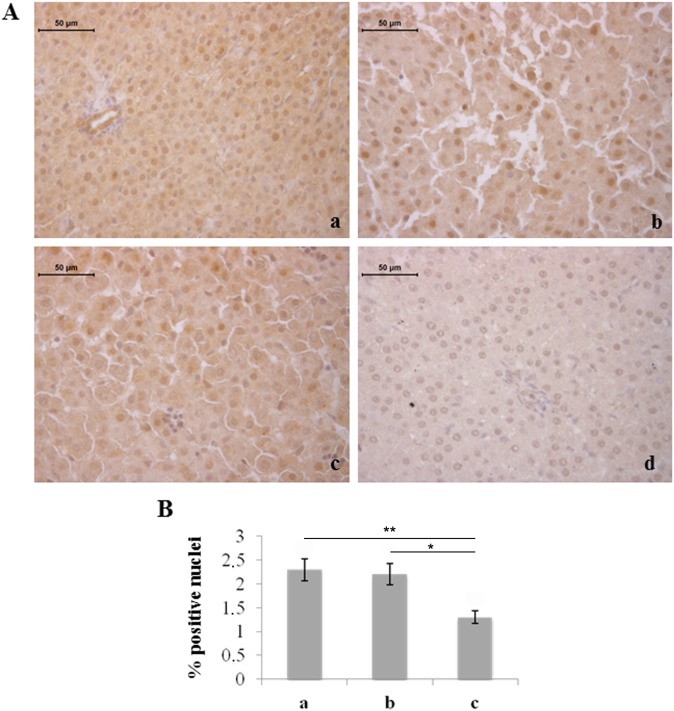
Severe hyperoxia decreases the number of hepatocytes immunostained for VEGF. A) Immunohistochemical detection of VEGF expression in neonatal rat livers exposed to moderate and severe hyperoxia. a) ambient air; b) 60% hyperoxia; c) 95% hyperoxia; d) negative control; scale bar: 50 µm, magnification 40x. B) Graphic representation of the percentage of VEGF positive nuclei (±SD); densitometric analysis determined by direct visual counting of ten fields for each of three slides per sample. * = p<0.05; ** = p<0.01.

**Figure 6:**
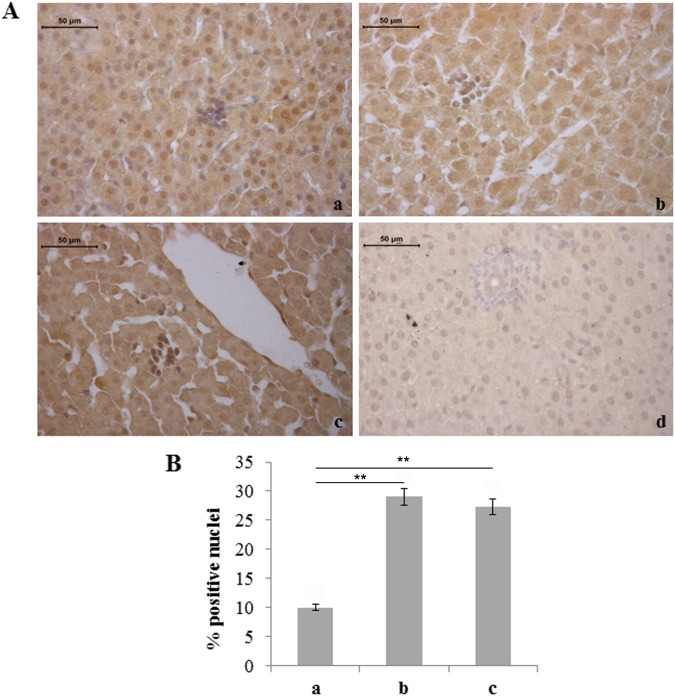
Moderate and severe hyperoxia increases VEGF expression in liver haemopoietic foci. A) Immunohistochemical detection of VEGF-positive haemopoietic cells in neonatal rat livers exposed to moderate and severe hyperoxia. a) ambient air; b) 60% hyperoxia; c) 95% hyperoxia; d) negative control; scale bar: 50 µm, magnification 40x. B) Graphic representation of VEGF-positive haemopoietic cells percentages (±SD); densitometric analysis determined by direct visual counting of ten fields for each of three slides per sample. ** = p<0.01.

We also considered molecules involved in haemopoiesis and remodeling of blood vessels. The number of hepatocytes immunostained for VEGF was found to be reduced in rats exposed to 95% hyperoxia with respect to the other two experimental groups (Fig. 5). Conversely, increased expression of VEGF, in terms of staining intensity and number of positive cells, was observed in the liver haemopoietic foci of rats exposed to moderate and severe hyperoxia (Fig. 6).

**Figure 7:**
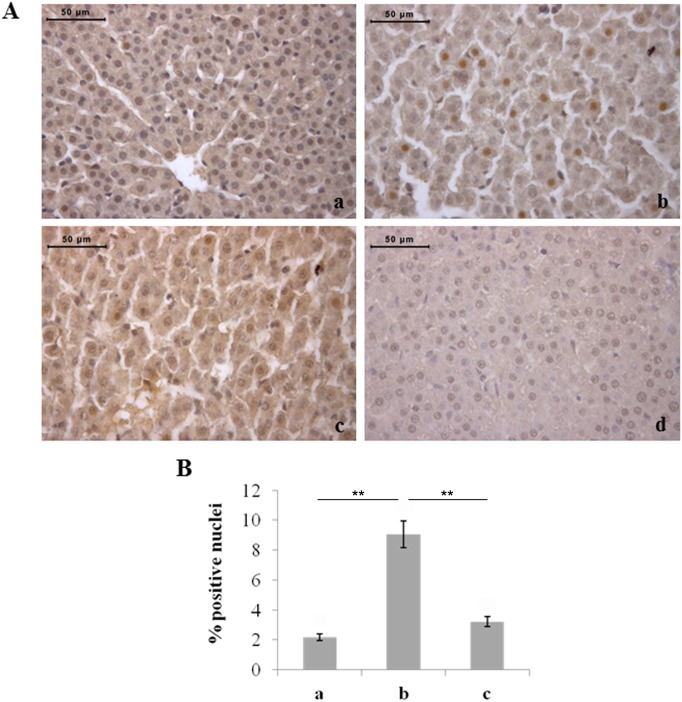
Moderate hyperoxia increases the number of HIF-1α-positive hepatocytes with respect to normoxia and severe hyperoxia. A) Immunohistochemical detection of HIF-1α expression in neonatal rat livers exposed to moderate and severe hyperoxia. a) ambient air; b) 60% hyperoxia; c) 95% hyperoxia; d) negative control; scale bar: 50 µm, magnification 40x. B) Graphic representation of the percentages of HIF-1α positive nuclei (±SD); densitometric analysis determined by direct visual counting of ten fields for each of three slides per sample. ** = p<0.01.

The expression of HIF-1α involved in oxygen homeostasis maintenance was also investigated. A statistically significant increase in the percentage of positive hepatocyte nuclei was found in rats exposed to 60% oxygen compared to the other two experimental groups (Fig. 7). Conversely, significant changes were not observed in the liver haemopoietic cells of the different experimental groups (data not shown).

**Figure 8:**
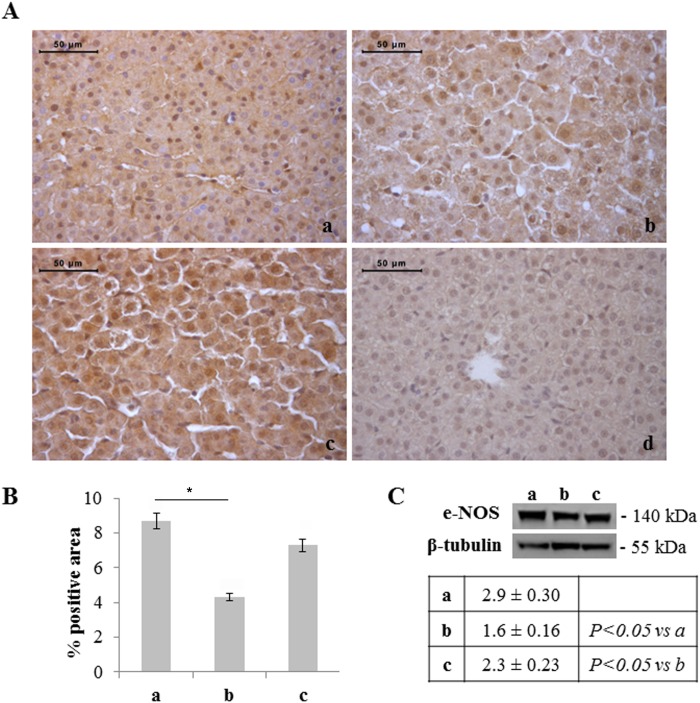
Moderate hyperoxia reduces hepatocyte eNOS expression with respect to normoxia and severe hyperoxia. A) Immunohistochemical detection of eNOS expression in neonatal rat livers exposed to moderate and severe hyperoxia. a) ambient air; b) 60% hyperoxia; c) 95% hyperoxia; d) negative control; scale bar: 50 µm, magnification 40x. B) Graphic representation of the percentage of eNOS positive area (±SD); densitometric analysis determined by direct visual counting of ten fields for each of three slides per sample. * = p<0.05. C) Western blotting analysis of eNOS expression in neonatal rats. Each membrane has been probed with anti β-tubulin antibody to verify loading evenness. The most representative out of three separate experiments is shown. Data are the densitometric measurements of protein bands expressed as mean values of Integrated Optical Intensity (IOI) (±SD).

**Figure 9:**
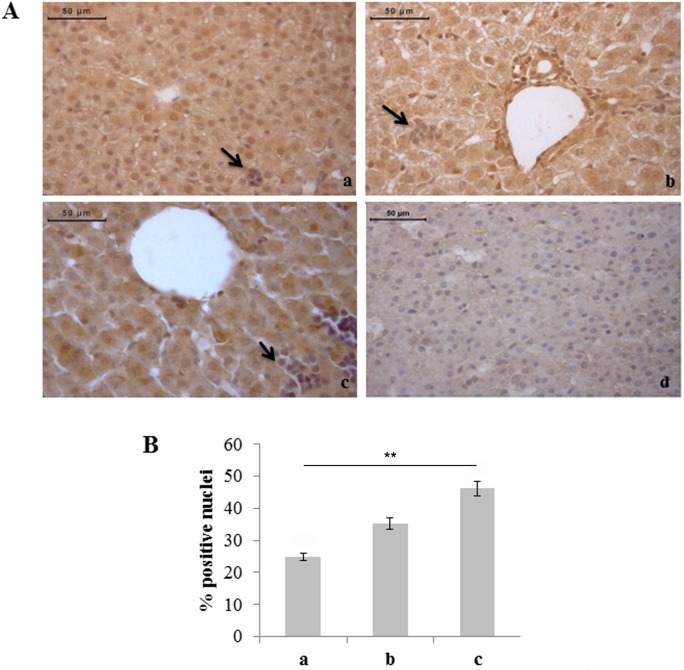
Severe hyperoxia increases eNOS expression in haemopoietic foci. A) Immunohistochemical detection of eNOS-positive haemopoietic cells (arrows) in neonatal rat livers exposed to moderate and severe hyperoxia. a) ambient air; b) 60% hyperoxia; c) 95% hyperoxia; d) negative control; scale bar: 50 µm, magnification 40x. B) Graphic representation of the percentages of eNOS-positive haemopoietic cells (±SD); densitometric analysis determined by direct visual counting of ten fields for each of three slides per sample. ** = p<0.01.

As it regards the expression of eNOS, a decrease in its positivity was found in hepatocytes of rats exposed to moderate hyperoxia with respect to normoxia (Western blot and immunohistochemistry) and severe hyperoxia (Western blot) (Fig. 8). Specific evaluation of haemopoietic foci, instead, showed a progressive increase in the number of positive cells with increased severity of hyperoxia, the percentage of immunostained cells being significantly higher in 95% hyperoxia than in normoxia (Fig. 9).

**Figure 10:**
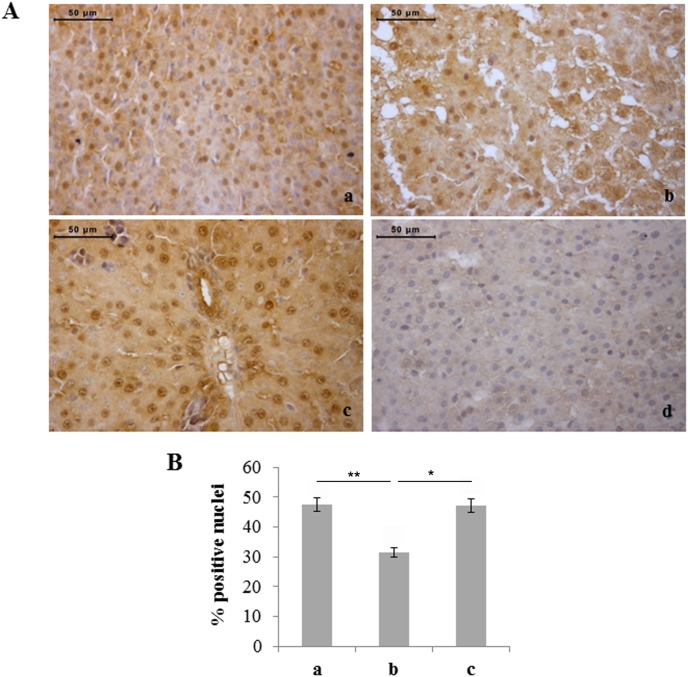
Moderate hyperoxia reduces the number of NF-kB positive hepatocytes with respect to normoxia and severe hyperoxia. A) Immunohistochemical detection of Nfκb expression in neonatal rat livers exposed to moderate and severe hyperoxia. a) ambient air; b) 60% hyperoxia; c) 95% hyperoxia; d) negative control; scale bar: 50 µm, magnification 40x. B) Graphic representation of the percentages of NF-kB positive nuclei (±SD); densitometric analysis determined by direct visual counting of ten fields for each of three slides per sample. * = p<0.05; ** = p<0.01.

Finally, NF-kB nuclear translocation was also studied through immunohistochemistry due to its role in the response to oxidative stress and development/differentiation of haemopoietic cells. The percentage of NF-kB positive hepatocyte nuclei was significantly lower in rats exposed to 60% hyperoxia than in controls and rats exposed to 95% hyperoxia (Fig. 10). Significant changes were not observed in the percentages of NF-kB positive liver haemopoietic cells of the different experimental groups (data not shown).

## Discussion

In the present work we evaluated the effects of postnatal hyperoxia exposure on rat liver with reference to both hepatocytes and hepatic haemopoietic cells, reporting differential effects on the two components. Markers of apoptosis and proliferation were considered, together with a series of factors previously found in other tissues to be involved in hyperoxic response.

There are papers in the literature reporting increased apoptotic cell index due to hyperoxia exposure in different tissues, such as lungs [33], brain [6], [34]–[37] and heart [25]. In the present work, statistically significant increase of apoptotic hepatocyte percentage was found in the livers of rats exposed to severe hyperoxia compared to unexposed ones. This finding indicates that hyperoxia may exert a noxious effect in the liver tissue of newborn rats, although only as a consequence of exposure to severe hyperoxia. Preceding works of our group involving analogous experimental conditions have also reported increased apoptosis in the heart [25] and dentate gyrus [19] in response to severe, but not moderate, hyperoxia.

As it regards haemopoietic foci, instead, a significant increase in the percentage of apoptotic cells was not found. Conversely, we observed a paradoxical response of liver hemopoiesis to hyperoxia, foci of liver haemopoiesis being larger and more numerous in rats exposed to severe hyperoxia with respect to controls. This finding may be interpreted in different ways. For instance, it could be a response to hyperoxia-induced haemolytic reactions, as exposure of rats to 80% hyperoxia for 11 days has been reported to increase haemolysis and decrease haemoglobin concentration [38]. Otherwise, it could be intriguing to hypothesize a direct effect of hyperoxia on haemopoietic stem cells. We have previously found, for instance, that both moderate and severe hyperoxia may induce a proliferative response in the main sites of postnatal neurogenesis, the subventricular zone and dentate gyrus [19]. Some mechanisms involved in hypoxia-induced increase in haemopoiesis could be paradoxically in common with hyperoxia-induced changes.

In the present paper, we also evaluated the extracellular matrix in order to find possible signs/mechanisms of liver damage and consider a possible role of its remodeling in the haemopoietic response. A decrease in the reticular fiber content was found in the liver of rats exposed both to moderate and severe hyperoxia, suggesting a possible role of changes in the organization of the local extracellular matrix.

MMPs, collectively called matrixins, are a family of proteases which specifically participate in remodeling and turnover of extracellular matrix components [39]. Specifically, MMP-9 is a member of the gelatinase family, also including MMP-2, which is known to be highly expressed in damaged and regenerating livers [40]–[41]. MMP-9 expression has been found to be modified in lung following hyperoxia [42]–[43]. MMP-9 plays an important role in the structural changes consequent to oxygen-induced lung injury in newborn mice [42]. Increased expression of MMPs by macrophages has also been associated with lung emphysema [44]. On the basis of these data, we also evaluated MMP-9 expression in the liver of rats exposed to hyperoxia and we observed an increased expression of MMP-9 in livers exposed to severe hyperoxia compared to moderate hyperoxia-exposed and unexposed ones. Thus, it may be hypothesized a role for upregulation of MMP-9 expression in the hyperoxia-induced remodeling of extracellular matrix.

VEGF may also play a key role in hyperoxia, specifically as a survival factor in oxidant injury [45]. Both increases and decreases in VEGF content have been reported in lung due to hyperoxia exposure, as a consequence of different experimental conditions (degree of hyperoxia, age and species of the animals, recovery periods) [45]–[48]. For instance, we previously reported decrease of VEGF expression in the lung of newborn rats exposed to 60% hyperoxia for the first 14 postnatal days [22] but few experimental data are available about hyperoxia-induced changes in VEGF expression in other tissues than lungs. VEGF and VEGF receptor 2 have been reported to be decreased in the brain of adult mice exposed to 50% hyperoxia for up to 3 weeks [31]. In a previous study of our group addressing the effects of hyperoxia on the heart (same experimental conditions of the present work), we reported a decrease and an increase of VEGF protein content in moderate and severe hyperoxia, respectively [24]. In liver, we found a different kind of change in VEGF expression: a reduction in the number of VEGF-positive hepatocytes was found in rats exposed to 95% hyperoxia with respect to the other two experimental groups. Thus, hyperoxia exposure showed an additional noxious effect on hepatocytes consisting in impairment of VEGF expression.

Investigation of VEGF expression was also particularly intriguing regarding haemopoietic cells as VEGF controls hematopoietic stem cell survival and proliferation through an internal autocrine loop mechanism [49]. Consistently with a role of VEGF in liver haemopoiesis induction, in our study we observed an increased expression of VEGF, in terms of staining intensity and number of positive cells, in the liver haemopoietic foci of rats exposed to moderate and severe hyperoxia. In the present study, anti-VEGF immunostaining also involved the nuclear compartment. This is consistent with other reports [50]–[51] and may be particularly important for the haemopoietic cells which are internally activated by VEGF [49]. Thus, cell specific activation of VEGF synthesis may be considered one of the mechanisms involved in the hyperoxia-induced haemopoietic response.

The expression of VEGF may be transcriptionally activated by HIF-1α [52] and previous study have shown that HIF-1α, although the major transcriptional factor involved in response to hypoxia [53], may also be activated by hyperoxia [24], [31], [54]. In the present work, however, HIF-1α immunostaining was not correlated with anti-VEGF positivity in hepatocytes. An increase in the percentage of anti-HIF-1α stained hepatocytes was found only in rats exposed to 60% hyperoxia. An increased expression of HIF-1α in haemopoietic cells would have possibly explained the upregulation of VEGF synthesis but changes were not found between the different experimental conditions. However, it must be considered that VEGF expression may also be activated by other alternative signaling pathways and regulators, such as PGC-1α [55], which could be investigated in the future.

The expression of eNOS was also evaluated as hyperoxia effects on the NOS expression and function have been reported [22], [24], [56]–[60] and NO is widely interrelated with the other factors considered. eNOS may be activated by VEGF or by stimuli such as hemodynamic shear stress and modified oxygen supply. It mediates, through NO production, vasodilatation, angiogenesis and increased vascular permeability [61]. Differential changes in eNOS expression have previously been reported in response to hyperoxia in various tissues. For instance, in a previous study of our group, the eNOS protein level was increased in the lung of newborn rats exposed to 60% hyperoxia for the first 14 postnatal days [22]. Conversely, eNOS protein content was decreased in the heart tissue of newborn rats exposed to 60% and 95% hyperoxia for the first 14 postnatal days [24] and the protein levels of eNOS and iNOS have been reported not to be modified in the liver of six-week-old mice exposed to >95% O_2_ for 72 or 96 h [11]. In the present study we also found different responses in hepatocytes and haemopoietic cells. In hepatocytes, eNOS expression was selectively reduced in moderate hyperoxia. In the liver tissue, the changes in eNOS and MMP9 expressions are similar and may be partially explained by previously reported finding that eNOS is essential for the efficient early induction of MMP-9 in damaged liver [62]. In haemopoietic foci, eNOS expression was progressively increased with increased degree of hyperoxia, reaching significance in severe hyperoxia with respect to normoxia. Thus, eNOS expression, directly induced by hyperoxia or indirectly activated by VEGF, could also play a role in hyperoxia-induced liver haemopoietic response. The hyperoxia-induced increase in eNOS expression in haemopoietic cells may be correlated with VEGF expression, as autocrine VEGF in acute myeloid leukemia cells has been reported to produce NO, through eNOS activation [63]. NO is also known to regulate haemopoiesis and stimulate cell growth in acute myeloid leukemia cells [63].

In the present study we also evaluated NF-kB nuclear translocation through immunohistochemistry, as NF-kB is involved in the response to oxidative stress [64] and it is known to play a role in development and differentiation of hemopoietic cells [65]–[66]. The percentage of NF-kB positive hepatocyte nuclei was significantly lower in rats exposed to 60% hyperoxia than in controls and rats exposed to 95% O_2_, whereas statistically significant differences were not found between normoxia and severe hyperoxia. An analogous finding was previously found in heart samples from the same experimental conditions, with decrease and increase of the percentage of NF-kB positive myocardial cell nuclei in moderate and severe hyperoxia, respectively [25]. Conversely, in the present study, significant differences were not found in NF-kB nuclear immunostaining of hemopoietic cells of the different experimental groups, although further studies will be needed to exclude a role for this factor in hyperoxia-induced changes in liver haemopoiesis.

In conclusion, our study showed different effects of hyperoxia on hepatocytes and haemopoietic cells, with growth factors and intracellular mechanisms being differently involved. Postnatal hyperoxia shows detrimental action on hepatic tissue, with increased hepatocyte apoptosis, increased MMP-9 expression and decreased reticular fiber content. Decreased VEGF expression may also play a role in severe hyperoxia whereas some other changes have been found to mainly involve response to moderate hyperoxia, such as increased HIF-1α expression, and decrease expression of eNOS and NF-kB. Conversely, postnatal hyperoxia exposure increases liver haemopoiesis and upregulates VEGF and eNOS expression. Thus, it may be hypothesized that hyperoxia stimulates proliferation of haemopoietic cells through VEGF and/or eNOS, and these findings may put further questions on the modulation of stem cell proliferation by changes in O_2_ concentrations.
